# Editorial: Ethnic differences in children in the clinical manifestation of infection with SARS-Cov-2 and its variants

**DOI:** 10.3389/fped.2024.1484221

**Published:** 2024-10-15

**Authors:** Chun-Ting Lin, Kai-Sheng Hsieh

**Affiliations:** ^1^ Department of Pharmacy, Chung Shan Medical University Hospital, Taichung, Taiwan; ^2^ Structural/Congenital Heart Disease and Ultrasound Center, Children’s Hospital, China Medical University, Taichung, Taiwan

**Keywords:** SARS-CoV-2, COVID-19, multisystem inflammatory syndrome in children (MIS-C), human immunodeficiency virus, aseptic meningoencephalitis, acute necrotizing encephalitis, febrile seizures (FS)

**Editorial on the Research Topic**
Ethnic differences in children in the clinical manifestation of infection with SARS-CoV-2 and its variants

Since the end of 2019, the virus known as Severe Acute Respiratory Syndrome Coronavirus 2 (SARS-CoV-2) has rapidly spread globally, leading to a pandemic. Initially, attention was primarily focused on adults, particularly the elderly over 65 years old and patients with pre-existing conditions. In adults, the most notable manifestation of this infection has been pulmonary involvement, with clinical symptoms ranging from asymptomatic to mild respiratory symptoms, progressing to severe pneumonia, acute respiratory distress syndrome (ARDS), and multiple organ dysfunction (1).

However, increasing research has revealed that the impact of COVID-19 on children is particularly noteworthy, as they were initially thought to be less susceptible to severe outcomes compared to adults. It has been observed that pulmonary involvement in children can be more severe than in adults, and numerous case reports have highlighted the occurrence of multisystem involvement. Clinical treatment and management approaches for pediatric patients cannot solely rely on data derived from adult cases (2).

This editorial aimed to synthesize the latest research findings to explore the impact of COVID-19 on children, including conditions such as acute necrotizing encephalopathy (ANE), aseptic meningoencephalitis (ME), febrile seizures (FSs), and multisystem inflammatory syndrome in children (MIS-C). These findings underscore the need for heightened awareness among healthcare providers.

As the COVID-19 pandemic has progressed, different SARS-CoV-2 variants have led to varying clinical presentations. Initially, children infected with SARS-CoV-2 exhibited mild respiratory symptoms. However, in later stages of the pandemic, particularly in patients infected with the Delta and Omicron variants, more life-threatening non-respiratory complications have emerged (3, 4). When clinical symptoms closely resemble other diseases, it can be challenging to make an accurate diagnosis promptly.

## Multisystem inflammatory syndrome in children

Multisystem inflammatory syndrome in children (MIS-C) is a systemic inflammatory syndrome that may occur in children following infection with SARS-CoV-2. The early clinical presentation of MIS-C is quite similar to Kawasaki disease, often presenting with symptoms such as fever, conjunctival injection, and rash (5). MIS-C has been reported mainly in Western countries; however, the incidence of MIS-C appears to be lower in Asian regions (6). According to a previous study, among 137 patients with MIS-C, only 4 (2.5%) exhibited symptoms of posterior pharyngeal swelling. If such cases occur in Asian regions where the incidence of MIS-C is lower and the incidence of Kawasaki disease is higher, clinicians should assess the risk of MIS-C in their patients (7). Both conditions can cause inflammation in multiple organ systems, requiring different management approaches. Therefore, it is crucial for clinicians to accurately diagnose and differentiate these conditions, especially when rare clinical symptoms, such as retropharyngeal edema, are present.

A few case reports have noted that patients with MIS-C may present with retropharyngeal edema. For example, a case report from Japan (Suzuki et al.) described a previously healthy 9-year-old boy who developed severe retropharyngeal edema resistant to intensive antibiotic treatment. Enhanced CT imaging revealed typical findings of the disease. Throat swabs and blood cultures performed during the initial examination were negative; however, records indicated a COVID-19 infection 3 weeks prior to the onset of symptoms. The patient, aged nine, which is a common age for MIS-C onset, experienced rapid symptom resolution and normalization of laboratory data following intravenous immunoglobulin treatment.

This case, along with previous reports (8), suggests that early use of intravenous immunoglobulin may help prevent the progression of MIS-C and the occurrence of circulatory failure. Additionally, retropharyngeal edema might be used as a predictor for MIS-C. For febrile children with severe neck symptoms and retropharyngeal edema, if MIS-C is suspected, recent COVID-19 history should be assessed, and early intervention may prevent disease progression.

## The impact of HIV on COVID-19 outcomes in resource-limited regions

According to the research conducted by (Byamungu et al.), the occurrence of cytokine storms indicates immune dysregulation. If not promptly addressed, this condition can lead to significant morbidity and mortality, particularly in regions with limited medical resources and among children with human immunodeficiency virus (HIV) infection, such as in Africa.

Previous studies have shown that the incidence and mortality rates of COVID-19 among hospitalized children and adolescents in these areas are significantly higher than in non African regions (9). These risks are associated with being under 1 year of age and with selected non communicable comorbidities, such as chronic kidney disease, chronic lung disease, blood disorders, liver disease, and chronic neurological conditions.

Moreover, children living in regions with high HIV prevalence or those exposed to HIV face a higher risk of severe COVID-19. This underscores the severity of COVID-19 among children with underlying medical conditions. Additionally, the results indicate that children with a history of comorbid conditions face a higher risk of severe disease and mortality, with HIV exposure and younger age being significant predictors.

The impact of COVID-19 on healthcare support systems is substantial, particularly in developing countries where patients often have multiple comorbidities and where local healthcare and transportation resources are limited (10). As the pandemic continues, effective integration and utilization of medical resources by local governments will be crucial for addressing these challenges.

## COVID-19 diagnosis and challenges in Asian populations

According to a series of case reports from Asian populations (Chao et al.) a comprehensive overview of non-pulmonary complications arising from COVID-19 in children is provided, including aseptic meningoencephalitis, acute necrotizing encephalitis, and multisystem inflammatory syndrome. The review offers recommendations regarding host factors, immunopathogenesis, and prevention (Table 1).

**Table 1:** Host factors, immunopathogenesis, and prevention of non-pulmonary COVID-19 in children.

Non-pulmonary COVID-19	Aseptic meningoencephalitis	Acute necrotizing encephalitis	Multisystem inflammatory syndrome
Immunopathogenesis	1. Age-associated immature immunity	1. Race: Asians >Caucasians	1. Race: Blacks, Asians
	2. Low neutralizing Abs with higher viral load	2. Genes: HLA, RANBP2, CPT2, SLC19A3, SCN1A	2. Genes: TRBV11-2, etc., linked to Th1 cytokine storm:IL-12 p70, IFNγ, etc.
	3. Interruption of BBB	3. Metabolism linked to hyperinflammation	3. Autoimmune vasculitis
Prevention and immunotherapies	1. Prophylactic vaccines	1. Screen genetic variants of hyperinflammation	1. Early IVIG and pulse therapy
	2. Passive immunization via maternal vaccination	2. Mitochondrial cocktails	2. Anti-cytokine storm therapy or anti-thrombosis treatment

Abs, antibodies; BBB, blood–brain barrier; CPT2, carnitine palmitoyltransferase II; RANBP2, RAN binding protein 2; SLC19A3, solute carrier family 19 member 3; SCN1A, sodium voltage-gated channel alpha subunit 1; TRBV11-2, T cell receptor beta variable 11-2.

Furthermore, new findings have emerged from studies assessing the severity of the disease. According to a retrospective study by Jin et al., the combination of the neutrophil-to-lymphocyte ratio (NLR) and eosinophil count is useful for diagnosing severe COVID-19 in pediatric patients. Specifically, an NLR greater than 2.17 and a near-zero eosinophil count in routine blood tests may indicate severe COVID-19 infection. Clinicians may need to adopt more aggressive treatment strategies in such cases to reduce the risk of mortality.

However, the applicability of these results may be limited due to the single-center design and small sample size of the study. Future research with larger sample sizes is needed to validate these findings.

The impact of COVID-19 infection on children varies widely across different countries, regions, and even among different racial groups. While multisystem inflammatory syndrome in children (MIS-C) and neurological complications are the primary focus of this discussion, the emergence of various viral variants has introduced numerous new challenges in clinical diagnosis and treatment, including an increased risk of febrile seizures (Liu et al.).

As we approach the fifth year since the outbreak of the COVID-19 pandemic at the end of 2019, our understanding of COVID-19 remains incomplete. Continuing to research the pathophysiology of COVID-19 in children, particularly the mechanisms behind severe manifestations and the roles of genetic and environmental factors, remains a critical issue. Future public health strategies should continue to prioritize vaccination for children, as it remains a key intervention for preventing severe illness and its complications.
